# Hippocampus—Related Cognitive and Affective Impairments in Patients With Breast Cancer—A Systematic Review

**DOI:** 10.3389/fonc.2020.00147

**Published:** 2020-02-21

**Authors:** Xenia Peukert, Karen Steindorf, Sanne B. Schagen, Adrian Runz, Patric Meyer, Philipp Zimmer

**Affiliations:** ^1^Division of Physical Activity, Prevention and Cancer, German Cancer Research Center (DKFZ), Heidelberg, Germany; ^2^SRH Hochschule Heidelberg, Heidelberg, Germany; ^3^National Center of Tumor Diseases, Heidelberg, Germany; ^4^Division of Psychosocial Research and Epidemiology, Netherlands Cancer Institute, Amsterdam, Netherlands; ^5^Department of Psychology, University of Amsterdam, Amsterdam, Netherlands; ^6^Department of Performance and Health (Sports Medicine), Institute for Sports and Sport Science, Technical University Dortmund, Dortmund, Germany

**Keywords:** breast cancer, cancer/cancer treatment-related side effects, hippocampus, cognitive impairments, affective impairments

## Abstract

**Background:** Although improvements in medical treatment lead to a steadily rising survival rate of breast cancer patients (BCP), it is associated with a decrease in cognitive and affective function. The hippocampus, a brain region with a high influence on both cognitive and affective function, is increasingly becoming the focus of current research because of its high vulnerability to adverse direct (chemotherapeutic agents, endocrine therapeutic agents, and radiation) or indirect (stress and other psycho-social factors) treatment-related effects.

**Methods:** This systematic review analyses current data from literature combining hippocampus-related brain changes due to breast cancer treatment with associated cancer-related cognitive and affective impairments (CRCI/CRAI). The seven studies that met the inclusion criteria consisted of six cross-sectional studies and one longitudinal study.

**Results:** The study results indicate hippocampal differences across all types of treatment. Those differences include volume loss, deformation, and changes in functional connectivity. They are associated with CRCI, revealing executive function as well as working memory, episodic memory, and prospective memory as the most affected domains. Although an interaction between hippocampus-related brain changes, CRCI, and CRAI can be hypothesized, CRAI are less reflected in current research.

**Discussion:** More research including longitudinal assessments with better overall methodology is needed to fully understand the interaction between hippocampal alterations and both CRCI and CRAI due to breast cancer treatment.

## Introduction

### Rationale

The ongoing global demographic and related epidemiologic changes indicate an ever-increasing cancer burden with more than 20 million new cancer cases expected annually within the next decade (1). Breast cancer is the most frequent cancer type among women (2). Due to improvements in early detection and treatment (3, 4), breast cancer survivorship rates continue to rise steadily since the 1990s (5, 6). In this context, a focus on breast cancer survivors‘quality of life seems to be of particular importance due to a vast body of literature reporting about lingering treatment–related side-effects cancer survivors have to deal with (7, 8). Those impairments can even persist up to 15 years after the end of treatment (9), inhibit occupational reintegration (10), and have a considerable influence on the quality of life of those affected (11).

Thereby, an increasing number of studies focused on measuring cancer–related cognitive impairments (CRCI) in those patients over the last ten years (12). They revealed attention, processing speed, executive function, and working memory as the most affected domains (9, 13, 14). Recent literature also provides evidence for various types of cancer-related affective impairments (CRAI) (15). A current study indicates an estimated prevalence of 48.6% for the development of anxiety and a prevalence of 15% for the development of depression in breast cancer patients (BCP) during and after the course of medical cancer treatment (16). Furthermore, an association between both anxiety and depression and CRCI in BCP undergoing chemotherapy and endocrine therapy seems to be present in current research (17, 18).

Trying to reflect the origin of these changes more precisely, both CRCI and CRAI have been associated with specific structural brain changes, including the temporal cortices (19). In this context, one of the most intensively studied brain regions, in both animal and human studies, is the hippocampus (20, 21). Due to its strong connection with other brain regions, including higher cortical brain structures and the limbic system, it is estimated that the hippocampal formation serves as a large integrating organ, which encodes and consolidates memory content by transforming new information, received from multiple brain regions (22). As a part of the posterior medial system, the posterior hippocampus is connected to the parahippocampal cortex, retrosplenial cortex, anterior thalamus, mammillary bodies and the pre- and parasubiculum. Moreover, it is connected with components of the default mode network, which plays a role in memory retrieval and spatial cognition (23, 24). Overall, the integration of processed information across the two cortical systems, supporting different kinds of memory-guided behavior, seems to depend on the dentate gyrus and cornu ammonis region (CA) 3 (23). Furthermore, the anterior hippocampal subregions are a part of the anterior temporal system, which are preferentially connected to the amygdala (25), the lateral orbitofrontal cortex as well as the ventral temporopolar cortex (23). It is involved in the hypothalamic-pituitary-adrenal axis (HPA), the major stress system in the body, as well as the limbic prefrontal circuit (26, 27).

The hippocampus generally consists of several subregions forming the so-called hippocampal formation, including the dentate gyrus, the subiculum, and the cornu ammonis regions with the CA1–CA4 fields (25, 28, 29). The dentate gyrus is thereby particularly involved in the process of generating new neurons in the hippocampus throughout life, a process called neurogenesis (30, 31). This mechanism mainly regulates the maintenance of brain plasticity, memory, and learning (32, 33). Studies provide evidence that the rate of maturation and survival of these cells are influenced by environmental conditions (34, 35).

For example, there is a lot of discussion on the key role of treatment-related structural brain changes in the hippocampus and its consequences for memory processes (36) but also for emotion-related processes (37). Among different forms of medical cancer treatment, chemotherapy is especially associated with hippocampal volume decrease (20), reduced neurogenesis (38–40), and has been linked to an array of experienced cognitive impairments (41). Especially hippocampal neurogenesis seems to be highly vulnerable to chemotherapeutic treatment as well as other types of cancer treatment (42, 43). For example, radiotherapy and endocrine therapy have been equally linked to volume loss and reduced hippocampal neurogenesis (42, 43). Interestingly, studies repeatedly documented a reduction in hippocampal volume in stress related psychiatric disorders like Major Depression (44, 45). Although it remains unclear whether hippocampus-related brain changes cause depression and anxiety or whether affective changes (for example caused by a cancer diagnosis) impact hippocampal structure and function as well as overall cognitive capabilities. Current research results indicate an association between CRAI and CRCI in BCP undergoing chemotherapy (17, 18).

### Objectives

A comprehensive theory revealing the underlying mechanisms of hippocampus-related brain changes due to cancer and its treatment is not present, even though current research and implications from animal studies indicate its role in causing CRCI. The aim of this systematic review is to elucidate the current knowledge on the impact of medical cancer treatment on the hippocampus and potential associations with CRCI and CRAI in BCP, investigated through specific brain imaging as well as neuropsychological assessment.

### Research Question

What influence do different forms of medical breast cancer treatment have on the hippocampus and how do these changes contribute to affective and cognitive impairments?

## Methods

### Study Design

Due to the fact that the question forms an interface between the medical and psychological field of science, the two databases PubMed and PsycINFO were searched for relevant literature in November 2018, after registering the systematic review in PROSPERO (ID: 117173). The search string used to perform this review was designed following the PICO (population, intervention, comparison, outcome) method (46). It is described as most effective for an overall comprehensive search (47, 48). In terms of formulating a well-focused question relevant to patient care as basis for any review (49), databases were searched regarding hippocampus-related brain changes induced by chemotherapy, endocrine therapy, and/or radiotherapy and their impact on CRCI and CRAI in BCP.

### Search Strategy and Data Extraction

The following key words and MeSH terms were used: “chemotherapy,” “cancer treatment,” “radiotherapy,” “hormone therapy,” “endocrine therapy,” AND “hippocampus,” “hippocampal,” “dentate gyrus,” “neurogenesis,” AND “breast cancer,” “breast tumor,” “breast carcinoma,” “breast neoplasm,” “mammary tumor,” AND “depression,” “mood,” “fatigue,” “affective,” “cognition,” “cognitive,” “impairment.” Additionally, the search was restricted to studies conducted within the last 10 years in order to appropriately represent the current status of research. Using this search string and the restriction concerning the publication date, 68 results were found after sorting out those studies occurring in both databases.

**Figure 1:**
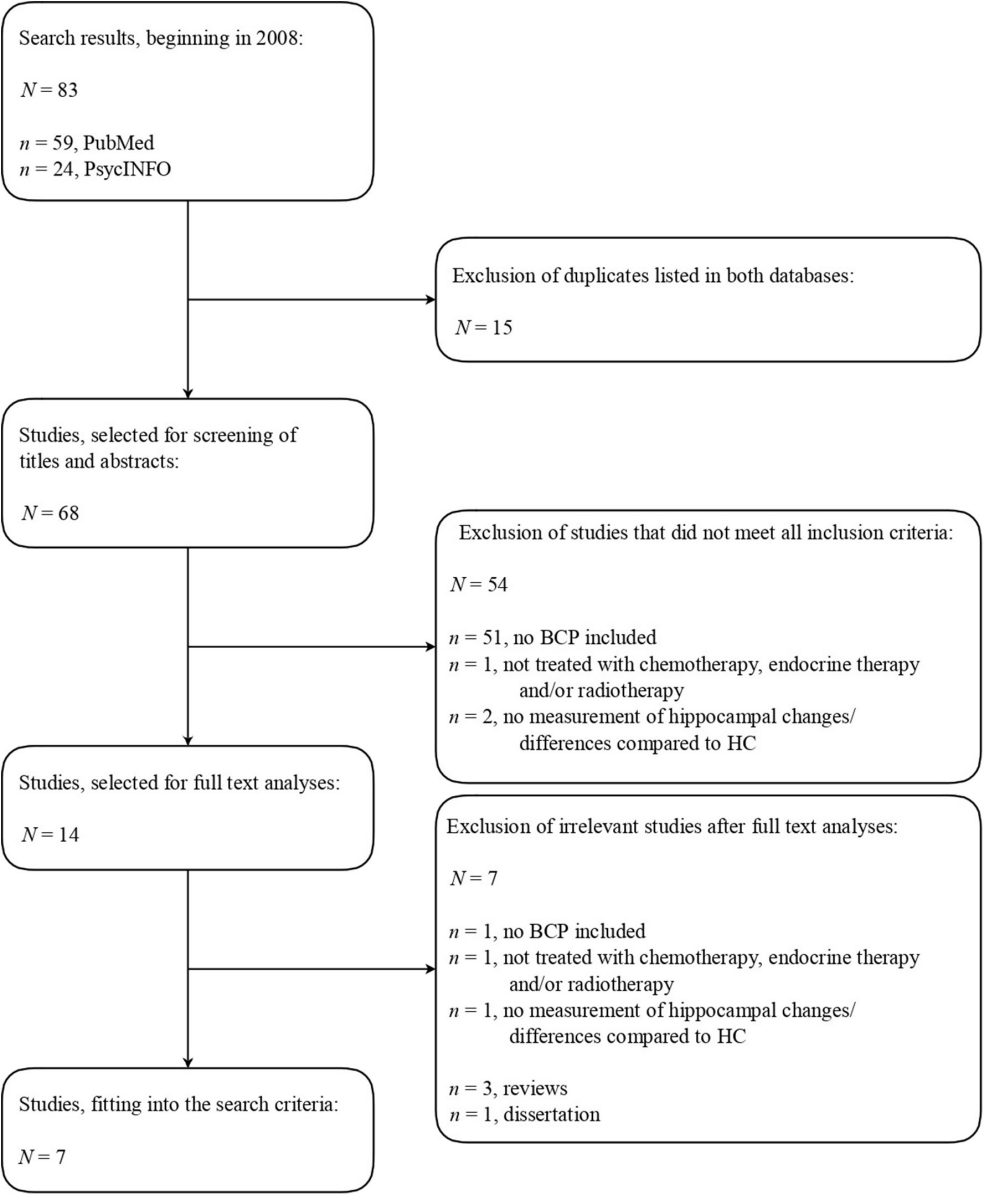
Literature search strategy.

During the initial screening of titles and abstracts, studies were included which met the following inclusion criteria: (a) the studies involved BCP (no animal studies); (b) treated with chemotherapy, endocrine therapy, and/or radiotherapy; (c) measurement of hippocampus-related brain changes/differences compared to healthy controls (HC); (d) neuropsychological tests; and (e) abstracts written in English. The detailed literature search strategy is shown in Figure 1.

## Results

### Study Selection and Characteristics

The initial screening showed that 54 studies had to be excluded in view of the fact that they did not match all inclusion criteria. In this process, animal studies have been deliberately excluded to ensure methodological comparability. Even though studies conducted with animals (40, 50) appropriately investigate crucial structural brain changes caused by the breast cancer treatment and are useful to discuss the study results conducted with humans, CRCI were not measured in a comparable way.

After full text screening of the remaining 14 studies, four studies were excluded because they did not meet all of the inclusion criteria. One study was excluded because it did not involve BCP, two studies because treatment was not specified and one study because measuring hippocampus-related brain changes was not involved. Moreover, three reviews were excluded from analysis after being screened for potential original data and one dissertation was excluded since studies indicate that dissertations rarely influence the conclusions of reviews (51).

Following this, data on authorship and publication year, study population, study design, hippocampus-related differences, parameters indicating differences in cognitive and affective function as well as correlations and additional findings were extracted from each study, as shown in Table 1.

**Table 1:** Human studies.

**References**	**Groups**	***n***	**Age (year) Mean ±*SD***	**Status of treatment**	**Study design**	**Hippocampus-related differences/changes (cHC)**		**Parameters indicating differences in cognitive/affective function (cHC)**		**Correlations and additional findings**
Bergouignan et al. (52)	BCP HC	16 21	48.73 (4.95) 47.68 (5.31)	“Standard treatment“, in remission since 18 months Ø 16.4 weeks of CT Ø 5.8 weeks of RT Ø 39.27 months in remission [*n* = 4 ET]	Cross-sectional study	Hippocampal volume Total Total anterior Left anterior Right anterior Total posterior Left posterior Right posterior	(↓8%) (→) (→) (→) (↓11%) (↓) (→)	EAMR (TEMPau task) Depression (MADRS) [Exclusion criterion MADRS score >10]	(↓20%) (↑)	EAMR score was predicted by the group/volume of posterior hippocampus and interaction of group by volume of posterior hippocampus
Kesler et al. (53)	BCP HC	42 35	54.6 (6.5) 55.5 (9.3)	Surgery and CT Ø 4.8 years off-treatment [*n* = 29 RT] [*n* = 22 ET]	Cross-sectional study	Hippocampal volume Left Right [*N* = 5 *n* = 2 BCP *n =* 3HC]	(↓) (→)	Verbal memory (HVLT-R) Subjective memory functioning (MMQ) Depression (CAD)	(↓) (↓) (→)	Association between cytokine levels and left hippocampal volume in BCP Association between verbal memory performance and cytokine levels/hippocampal volume in both groups
Chaddock-Heyman et al. (54)	BCP HC	29 27	55.55 (1.48) 55.44 (2.13)	Surgery and CT/RT Ø 17 months off-treatment [*n* = 11 RT *n* = 7 CT *n* = 11 RT and CT]	Cross-sectional study	Hippocampal volume Total Total anterior Left anterior Right anterior Total posterior Left posterior Right posterior	(→) (→) (→) (→) (→) (↓) (→)	Spatial memory (memory “swap” errors) Cognitive function (MMSE) [Exclusion criterion MMSE score ≤ 23]	(→) (→)	Positive correlation between CRF and hippocampal volume in BCP No differences in hippocampal volume between higher fit BCP and HC Smaller left posterior hippocampal volume in lower fit BCP compared to lower fit HC Large effect for the difference in total hippocampal volume between lower fit BCP and HC
Chen et al. (55)	BCP HC	31 32	44.97 (4.56) 43.66 (4.66)	Treated with tamoxifen for at least 24 months Ø 40.45 months [*n* = 12 RT]	Cross-sectional study	Whole-brain FC FC of the right DLPFC with the right Hippocampus (↓)		General cognitive function (MoCa) Short term memory (DS) Processing speed (SCWT/TMT-A) General executive function (SIT/TMT-B) Working memory (2-back ACC/2-back RT) Depression (HAMD) Anxiety (HAMA)	(→) (→) (→) (↓) (↓) (→) (→)	Correlations between the functional connectivity strength of the right DLPFC with the right hippocampus and the ACC in the 1-back task, 2-back task/RT in the 2-back task
Perrier et al. (56)	BCP HC	20 27	53.95 (4.75) 56.44 (3.17)	T1: After surgery, before initiation of adjuvant therapy T2: One month after the end of chemotherapy T3: One year after the end of chemotherapy	Comparative longitudinal study	Gray matter volume at T2 in the left hippocampus	(↓)	Episodic Memory Verbal T1/T3 (ESR) Visual (BEM) Working memory (WAIS III) Executive function T3 (TMT/Verbal fluency) Processing speed (TMT) Depression (BDI) Anxiety (STAI-A and B) P-high > P-low at T2	(↓) (→) (→) (↓) (→) (→)	Larger anxiety at T2 was linked with a significant atrophy in the left posterior hippocampus in P-high compared to P- low Performance on neuropsychological tests was not directly related to hippocampal atrophy
Cheng et al. (57)	BCP HC	34 34 31	- 52.00 (8.48) 50.61 (8.32)	Before CT (CB) After CT (CC)	Cross-sectional study	Bilateral hippocampal FC Hippocampal FC in the frontal and parietal cortex, precuneus, PCC, and cerebellum Hippocampal FC in the right parahippocampus and left temporal pole	(↑) (↓)	cHC & (cCB) Cognitive function (MMSE) Executive function (VFT) Working memory and attention (DS) EBPM TBPM	(↓) (↓) (↓) (↓) (↓)	Connectivity between right hippocampus and bilateral precuneus was negatively correlated with DS performance Connectivity between left hippocampus and PCC.B and left MOG was negatively correlated with VFT scores in CC patients Left hippocampus and left FFA connectivity were negatively correlated with EBPM scores Connectivity between left hippocampus and Cbm.R was negatively correlated with EBPM/TBPM scores in BCP
Apple et al. (58)	BCP HC	16 18	37.93 (5.20) 27.17 (4.08)	CT within 18 months prior to the study and ET Ø 14,43 months off-treatment	Cross-sectional study	Hippocampal deformation Total Right Left Hippocampal volume Total	(↑) (↑) (→) (↓)	NIH Toolbox Cognition Battery Episodic memory Attention Processing speed Executive function Language Neuro-QoL General cognitive concerns Executive function concerns Anxiety Depression Fatigue	(↓) (→) (→) (→) (→) (↑) (→) (→) (→) (→)	

*BCP, Breast cancer patients; HC, Healthy controls; CT, Chemotherapy; RT, Radiotherapy; ET, Endocrine therapy; cHC, compared to healthy controls; →, no significance; EAMR, Episodic autobiographical memory retrieval; TEMPau task, Test Episodique de Mémoire du Passé autobiographique; MADRS, Montgomery-Åsberg Depression Rating Scale; HVLT-R, Hopkins Verbal Learning Test-Revised; MMQ, Multifactorial Memory Questionnaire Ability Scale; CAD, Clinical Assessment of Depression; MMSE, Mini-Mental-Status Examination; CRF, Cardiorespiratory fitness; CB, Before chemotherapy; CC, After chemotherapy; FC, Functional connectivity; PCC, Posterior cingulate cortex; cCB, compared to before chemotherapy; VFT, Verbal Fluency Test; DS, Digital span; EBPM, Event-based prospective memory; TBPM, Time-based prospective memory; PCC.B, Bilateral cingulate cortex; MOG, Middle occipital gyrus; FFA, Fusiform area; Cbm.R, Right cerebellum; Neuro- QoL, Quality of Life in Neurological Disorders; DLPFC, Dorsal lateral prefrontal lobe; MoCa, Montreal Cognitive Assessment Test; SCWT, Stroop Color and Word Test; TMT-A/B, Trailmaking Test A/B; SIT, Stroop Interference Test; ACC, Accuracy; RT, Reaction Time; HAMD, Hamilton Depression Rating Scale; HAMA, Hamilton Anxiety Rating Scale; ESR, Encoding Storage Retrieval; BEM, Batterie d'efficience mnésique; P-high/low, high/low level of education*.

As shown in Figure 1, six cross-sectional studies and one longitudinal study were chosen for further analysis due to fitting the inclusion criteria, comprising a total number of 381 individuals. Of these, 190 were BCP and 191 were HC. The selected studies were substantively sorted by collecting and comparing findings on hippocampal as well as cognitive and affective changes or differences between BCP and HC and their correlation, based on the extracted information shown in Table 1.

### Synthesized Findings

#### Structure- and Connectivity-Related Hippocampal Changes or Differences in BCP Compared to HC

All seven studies illustrated structure- and connectivity-related hippocampal changes or differences compared to HC, including hippocampal volume loss (52–54, 56, 58), hippocampal deformation (58) as well as changes in bilateral hippocampal (57) and whole-brain functional connectivity (55). Reduced hippocampal volume was reported in most of the studies, referring mainly to the left hippocampus (53, 54) and posterior regions (52, 54). Bergouignan et al. (52) found a posterior hippocampal volume reduction of 11% for BCP in remission from “standard treatment,” which included tumorectomy, chemotherapy and radiotherapy, compared to HC. By assessing hippocampal gray matter volume in BCP before (T1), 1 month after (T2), and 1 year after chemotherapeutic treatment with fluorouracil, epirubicin, cyclophosphamide and docetaxel (T3), Perrier et al. (56) also provided insides into time effects. Gray matter volume reduction was found in the left hippocampus 1 month but not 1 year after chemotherapeutic treatment. Perrier et al. (56) also linked hippocampal atrophy to anxiety and education by showing that larger anxiety in highly educated BCP at T2 was linked with a significant atrophy in the left posterior hippocampus but not in lower educated BCP. Additionally, findings of Chaddock-Heyman et al. (54) highlight the role of individual differences in cardiorespiratory fitness (CRF) on hippocampus-related brain changes. Even though large differences were measured for lower fit BCP compared to HC, higher fit BCP did not differ in hippocampal volume compared to HC. Kesler et al. (53) also associated reduced left hippocampal volume in BCP with lower levels of IL-6 and higher levels of TNFα.

In contrast to hippocampal volume reduction, hippocampal deformation (meaning specific morphological abnormalities and differences in shape compared to healthy controls) in BCP who underwent chemotherapy and endocrine therapy were mainly observed in the right hippocampus after controlling for age compared to HC (58). In addition to structural differences, two studies measured differences in functional connectivity, including whole brain functional connectivity (55) and bilateral hippocampal network connectivity (57). Thereby, Chen et al. (55) found decreased functional connectivity of the Dorsolateral prefrontal cortex (DLPFC) with the right hippocampus in BCP treated with tamoxifen compared to HC. Additionally, Cheng et al. (57) found right hippocampus in BCP treated with tamoxifen compared to HC. Additionally, Cheng et al. (57) found chemotherapy compared to HC, including not only the frontal and parietal cortex but also the precuneus, posterior cingulate cortex, and the cerebellum. Decreased hippocampal functional connectivity in the left hippocampal network was present with the right parahippocampus and in the right hippocampal network with the left temporal pole.

#### Subjectively Experienced and Objectively Measured Differences in Cognitive Function

Within two studies, subjective assessments of cognitive function and memory ability was provided, including subjective memory function (53) and general cognitive concerns as well as executive function concerns (58). In this context, BCP, who underwent chemotherapy (cyclophosphamide or paclitaxel and doxorubicin; 5-fluorouracila and paclitaxel or methotrexate and cyclophosphamide; taxane, anthracycline, and cyclophosphamide) and endocrine therapy (tamoxifen), and HC did not differ regarding executive function concerns. However, subjective memory function was decreased in BCP after surgery and chemotherapy (53) and global cognitive concerns were increased in BCP after being treated with chemotherapy and endocrine therapy (58). In addition to subjective assessments, six studies objectively recorded differences in cognitive function in BCP compared to HC, including worsened verbal memory (Hopkins Verbal Learning Test-Revised) (53) and episodic memory performance (Picture Sequence Memory Test) (58) as well as declines in episodic memory retrieval (Test Episodique de Mémoire du Passé autobiographique task) (52), following various forms of breast cancer treatment (52, 53, 58). Beyond that, BCP, who have been treated with tamoxifen, performed significantly worse in tests evaluating general executive function and working memory tasks (55).

Cheng et al. (57) compared cognitive deficits of BCP after chemotherapy to BCP before treatment. They found a decrease in cognitive function, using the Mini Mental Status Examination (MMSE), in executive function, using the Verbal Fluency Test (VFT), and in working memory and attention, measured by digital span (DS) performance as well as event-based and time-based prospective memory (EBPM/TBPM). Additionally, Perrier et al. (56) assessed changes in episodic memory, working memory, executive functions as well as processing speed longitudinally with differing results. BCP performed significantly worse in episodic verbal memory retrieval (Encoding Storage Retrieval) at T1 (after surgery but before initiation of adjuvant therapy) and T3 (1 year after the end of chemotherapy) and showed lower performances than HC at T3 regarding executive function tasks, using the Trail Making Test (TMT) and Verbal Fluency Test (VFT). No differences between HC and BCP were found for visual episodic memory, using the Batterie d'efficience mnésique (BEM) as well as processing speed, using the TMT.

#### Correlations Between CRCI and Hippocampus-Related Differences

Overall, findings indicate that the mentioned differences in measured cognitive function can be associated with hippocampal regions, differing between left and right hippocampus as well as anterior and posterior regions within the hippocampus.

##### Left hippocampus

With regards to the left hippocampus, Kesler et al. (53) found associations between hippocampal volume reduction and cytokine levels (IL-6 decreased and TNFα increased) and diminished verbal memory performance assessed with the HVLT. By measuring bilateral hippocampal connectivity, Cheng et al. (57) linked worsened test results in tests measuring executive function (VFT) as well as EBPM and TBPM to changes in bilateral hippocampal functional connectivity. Thereby, connectivity between the left hippocampus, bilateral cingulate cortex, and left middle occipital gyrus was negatively correlated with VFT scores. Moreover, the left hippocampus and left fusiform area were negatively correlated with EBPM scores and connectivity between the left hippocampus and right cerebellum was associated with declines in EBPM and TBPM scores.

##### Right hippocampus

Two studies (55, 57) indicate that the functional connectivity between the right hippocampus and other brain regions is associated with working memory performance. In this context, correlations between the functional connectivity strength of the right dorsal lateral prefrontal lobe (DLPFC) with the right hippocampus and accuracy in the 1-back and 2-back task of the DS test and the reaction time in the 2- back task were measured (55). Moreover, connectivity between the right hippocampus and bilateral precuneus was negatively correlated with DS performance (57).

##### Posterior hippocampal regions

Posterior regions within the hippocampus were mainly associated with changes in episodic memory retrieval and declines in spatial memory performance. Bergouignan et al. (52) examined significant differences in episodic autobiographical memory retrieval (measured with TEMPau task) between BCP after “standard treatment,” including chemotherapy, radiotherapy and endocrine therapy, and HC, showing that BCP had significantly lower episodic memory retrieval than HC. Additionally, episodic memory score was predicted by the volume of the posterior hippocampus and the interaction of group by volume of the posterior hippocampus. Chaddock-Heyman et al. (54) compared higher and lower CRF in BCP to higher and lower CRF in HC but found no significant differences between BCP and HC in their self-constructed spatial memory task, even though the means indicate more “swaps,” errors made during reconstructing the relative positions of objects, in BCP. However, memory “swap” errors were related to reduced left posterior hippocampal volume in BCP.

A contradictory picture was only drawn by Perrier et al. (56), who reported a decrease in gray matter volume 1 month after chemotherapeutic treatment (epirubicin, fluorouracil, docetaxel, and cyclophosphamide) but no direct relation between performances on neuropsychological tests (ESR, BEM, WAIS III, TMT) and hippocampal atrophy.

#### Changes in Affective Function in BCP and Differences Compared to HC

Generally, results on differences in affective function must be considered with caution due to the fact that in six out of seven studies, to avoid potential confounding, BCP with a current or past history of psychiatric disorders were excluded (52, 53, 55–58). However, five of seven studies (52, 53, 55, 56, 58) included measuring changes or differences in affective function compared to HC. Five of seven studies recording depression scores by using the Montgomery Åsberg Depression Rating Scale (52), Clinical Assessment of Depression (53), Neuro-QoL (58), Beck Depression Inventory (56) and Hamilton Depression Rating Scale (55), reported no significant differences between BCP and HC. Only one study (52) found significantly higher depression scores in BCP in remission after “standard treatment,” including chemotherapy and radiotherapy, compared to HC. Besides, both studies measuring anxiety scores (55, 58) found no differences in BCP, treated either with endocrine therapy (55) or a combination of chemotherapy and endocrine therapy (58). Additionally, using the Neuro QoL, Apple et al. (58) recorded self-reported impairment information surrounding fatigue but found no differences between BCP and HC.

## Discussion

Only few human studies with predominately methodological limitations focused on linking those two research topics.

### Summary of Main Findings

The results on hippocampus-related consequences of cancer treatment are in line with the empirically substantiated assumption that the hippocampus is highly vulnerable during the course of cancer and its treatment (20, 21). In addition to chemotherapy, volume loss, and reduced hippocampal neurogenesis (42, 43) were present in studies equally focusing on radiotherapy (54), or endocrine therapy (55). Moreover, possible diverse CRCI due to hippocampus-related brain changes were recorded, reflecting the assumption that the effects of cancer treatment on the hippocampus result in declines in many tasks related to memory and learning processes (36, 52, 59, 60).

With regard to memory-related cognitive function, the studies indicate that impairments are evident in tasks measuring hippocampus-related executive function as well as working memory, episodic memory, and prospective memory tasks (52, 55–58). These results are particularly interesting since research results suggest that the memory processes involved are highly interrelated. For example, McCabe et al. (61) found out that correlations between episodic memory and either working memory capacity or executive function ranged between *r* = 0.73 and *r* = 0.90. They even advocate an underlying mechanism which they call executive attention. Impairments in executive function were measured in three out of four studies for BCP treated with chemotherapy, using the TMT-B/VFT56 and the VFT57, as well as for BCP treated with endocrine therapy, using the SIT and the TMT-B58, compared to HC. Thereby, Cheng et al. (57) found out that connectivity between the left hippocampus and bilateral cingulate cortex and the left middle occipital gyrus was negatively correlated with VFT scores in BCP.

Due to the fact that executive function generally includes control functions related to inhibiting prepotent responses, shifting mental sets, updating task demands, planning, working memory as well as cognitive flexibility (61), it is not surprising that a deterioration of the working memory was present in most of the studies. The study results further helped to provide an insight into the possible causes of impairments in working memory by linking impairments to changes in functional brain connectivity (55, 57). According to Cheng et al. (57), connectivity between the right hippocampus and bilateral precuneus was negatively correlated with DS performance, measuring working memory, and attention. As a part of the posterior medial system, the hippocampus is connected with the default mode network, which the precuneus is a part of (23), that plays an important role in memory retrieval processes (24). Additionally, Chen et al. (55) linked worsened working memory performance, measured by the 1-back and 2-back task, with decreased functional connectivity strength of the right DLPFC with the right hippocampus.

Attention processes are mainly related to the functioning of the working memory, being included in the study by Cheng et al. (57) and measured individually by Apple et al. (58), and the prefrontal cortex is especially related to attention-based processes (80). Contrary to the results of Cheng et al. (57), Apple et al. (58) found no differences in attention, included in the NIH Toolbox Cognition Battery, between BCP treated with chemotherapy and endocrine therapy and HC. Interestingly, BCP treated with tamoxifen and HC did not differ in DS performance (55). Also, measuring digital span performance longitudinally, Perrier et al. differ in DS performance (55). Also, measuring digital span performance longitudinally, Perrier et al. impairments in spatial memory were recorded by Chaddock-Heyman (54), focusing on BCP treated with radiotherapy and/or chemotherapy on average 17 months off-treatment, even though spatial cognition is equally linked to the default mode network in current research results (24).

The studies involved indicate that besides working memory, episodic memory is highly affected by breast cancer treatment. Two studies including episodic memory performance found that it was significantly worsened in BCP in remission from “standard treatment,” containing all types of treatment (52), as well as in BCP treated with chemotherapy and endocrine therapy (58) compared to HC. Thereby, Bergouignan et al. (52) provided an insight into the extent and the causes of episodic memory impairments due to hippocampus-related brain changes. They did not only find out that the BCP group had 20% less access to episodic autobiographical memory retrieval than HC, but that episodic autobiographical memory retrieval score was predicted by the group and volume of the posterior hippocampus as well as the interaction of group by volume of the posterior hippocampus. These results can also be well-integrated into the current knowledge on the role of the posterior hippocampus in memory processes as it forms a part of the posterior medial system that plays a role in recollection and episodic memory (23). Because memory-related information from both systems is integrated in the dentate gyrus and the CA3 region (23), it is not surprising that those impairments have been linked to disrupted neurogenesis in mouse models (38, 40, 59). Furthermore, the study of Cheng et al. (57) included measuring both EBPM and TBPM. Thereby, BCP treated with chemotherapy performed significantly worse in tasks related to EBPM as well as TBPM compared to HC and compared to BCP before treatment. Reasons may be found in the connectivity of the left hippocampus with other brain regions. Thereby, the left hippocampus and left Fusiform area (FFA) connectivity were negatively correlated with EBPM scores and the connectivity between the left hippocampus and the right cerebellum was negatively correlated with both EBPM and TBPM scores in BCP (57). Interestingly, results by Perrier et al. (56) indicate, that declines in episodic memory in BCP compared to HC are already present before treatment since BCP showed lower performances in verbal memory retrieval, measured by Encoding Scoring Retrieval, both before initiation of adjuvant chemotherapy and 1 year after the end of chemotherapy. Also, even though differences between BCP and HC were found for verbal episodic memory, no significant differences were found for visual episodic memory, using the BEM (56). This suggests that other factors may also have an impact on CRCI and that there may be a difference in the vulnerability of different types of episodic memory to treatment- related effects.

Recent research results also indicate that CRAI as a result of hippocampus- related impairments could represent one influential factor by pointing to the interacting effects between cognitive and affective functions and brain structural changes. For example, Perrier et al. (56) found out, that larger anxiety scores were linked to significant atrophy in the left posterior hippocampus in highly educated BCP but not in less educated BCP. Nevertheless, even though most of the studies included the recording of depression (52, 53, 55, 56, 58) and anxiety scores (55, 56, 58), CRAI did not play a central role in the studies involved. For instance, measuring fatigue, one of the most common symptoms that can even persist up to 10 years after the end of treatment (62, 63), was only included in one study (58). Despite one study (52), no differences in affective function were found between BCP and HC although different tests were used and different treatment types were included. These results correspond to the hippocampus-related results to the extent that the volume reduction due to the breast cancer treatment mainly seems to have an influence on posterior parts (52, 54). This is in line with the assumption of affective impairments being more closely related to changes in anterior parts of the hippocampus due to connections with the amygdala (25), HPA axis and the limbic prefrontal circuit (26, 27). However, current research results indicate that CRAI would have been likely since neurogenesis in the dentate gyrus plays a role in buffering stress responses and depressive behavior by modulating the HPA axis (64). Moreover, Kesler et al. (53) found out that there is an association between cytokine levels and left hippocampal volume and a link between inflammation and depression has been increasingly suggested (65, 66). This is in line with current research results linking pro-inflammatory cytokines to synaptic dysfunction and neuronal death, particularly focusing on neurogenesis impairment in the dentate gyrus due to several direct and indirect effects, including the death of neural progenitor cells as well as limiting effects on neuronal differentiation (67). An association between lower levels of IL-6 with lower left hippocampus volume reported by Kesler et al. (53) were in contrast to previous findings, suggesting an inverse relationship between IL-6 levels and hippocampus volume in healthy adults (53). As an explanation for this contradictory finding, Kesler et al. referred to an altered pattern of influence for IL-6 in patients with a history of various illnesses. Furthermore, IL-6 seems to fulfill a role as a pro- as well as an anti-inflammatory agent following brain injury, which is indicative of a complex mechanism regulating IL-6 levels in patients with cancer and during/after its treatment (53).

Comparing results of the evaluated studies with recent findings on depression and anxiety in breast cancer survivors (BCS), a systematic review from Carreira et al. (68) found 33 studies reporting more depression in BCS compared to women without cancer (with 19 studies being statistically significant) and 17 studies reporting more anxiety (with 11 studies being statistically significant). The reasons for increased depressive symptoms in BCS seemed to be comparable to those in the general female populations, including: lower rates of social and psychological support, lower socio-economic status as well as impact on lifestyle and relationship (68). Interestingly, a phenomenon called “posttraumatic stress growth,” which goes along with feelings of improved empathy, closer relationships and great appreciation of life is reported in about 60% of BCS and might be a reason why symptoms of anxiety and depression are not persistent in some subgroups of BCS (68). Although this review provides compelling evidence of BCS being at increased risk for development of depression and anxiety, comparable to the studies used in our review, certain limitations were mentioned by the authors. Those limitations included the cross-sectional study design, low power, selection bias of participants, information bias, no control for confounding factors such as age and socio-economic status and methodological limitations of how depression and anxiety are assessed in various studies.

### Methodological Limitations

First of all, the cross-sectional design of six of the seven studies has to be seen within its limitations. The fact that most of the studies focused on CRCI and brain structural changes after the end of treatment has to be discussed since current research results support the assumption that CRCI are already observed before treatment (56, 69). In this context it also has to be mentioned that most of studies did not precisely differentiate between the types of breast cancer treatment or the chemotherapeutic agents the BCP had been treated with (52–54, 56–58). Including pre-treatment baseline assessments, comparable to the study design by Perrier et al. (56), and a differentiation between the treatment types may contribute to a better understanding of side effects attributable to the treatment (70).

Using expensive imaging techniques to properly measure structural brain changes makes it difficult to guarantee high sample sizes. It can therefore not be excluded that the relatively small sample sizes may have reduced statistical power in detecting smaller effects (53). It also must be taken into account that BCP often belong to a relatively old population (71) and that the hippocampus and especially the dentate gyrus are influenced by aging processes during the course of life (72, 73).

Moreover, only two studies included subjective measures (53, 58) even though subjective complaints are considered to be the heart of the CRCI problem (74). Objective measures of CRCI ranged from focusing on a single task (52) to including test batteries covering multiple cognitive domains (58). Thereby, even though focusing on just a few or even just one facet of cognitive function contributes to providing concrete information on one point of interest, a comprehensive picture of the complex interrelationships may not be derivable. The influence of affective factors on cognitive function is also not fully understood, mainly because CRAI were often used as an exclusion criterion in the studies involved. This is not only problematic because CRAI seem to play a major role in the treatment-caused side effects (15) but also because latest findings indicate that psychological variables do contribute to CRCI and also to hippocampus-related brain changes BCP have to deal with (56, 75).

### Limitations of This Review

The results should be considered within the context of the limitations of the present review. First of all, even though study selection was performed by two independent reviewer, selection bias cannot be fully precluded. Secondly, it was based on the overall data retrieved from the literature, so the stated limitations of the studies must therefore also be regarded as limitations of the present work. It cannot be completely ruled out that errors have occurred or wrong conclusions have been drawn trying to link and compare studies using different experimental designs, chemotherapeutic agents, patient populations (12), and tests measuring hippocampus-related changes and resulting CRCI and CRAI differently. Thereby, as already emphasized, comparisons, and connections between studies conducted with animals and those with humans must also be drawn with caution.

### Future Need for Research and Implementation of Clinical Study Results in Medical Routine

The studies involved in this review indicate that a highly relevant but yet understudied field of research revolves around the question of how hippocampus-related brain changes due to breast cancer treatment result in CRCI and CRAI. In order to get a deeper understanding of the complex interrelationships further research is required. For this purpose, future research should include baseline assessment not only of the cognitive and affective abilities but of the condition of the hippocampus pre-treatment by using longitudinal assessments and adjusting for age and patients abilities. This is also supported by the fact that breast cancer treatment relies on a combined treatment approach (76) and a differentiation of the different types of treatment could provide information on whether they lead to different consequences and whether interaction effects occur.

Since both animal and human studies indicate that the dentate gyrus is of decisive relevance to both CRCI and CRAI in BCP (40, 59, 60, 64, 77), it would be interesting to include tasks in future research which address functions that are specifically associated with the dentate gyrus. The findings further plead for a focus on tasks measuring executive function, working memory, episodic memory as well as prospective memory because they seem to be those cognitive domains being particularly influenced. Therefore, research results could be replicated using larger sample sizes.

Besides measuring CRCI, a field of research that is not reflected in current literature is the effect of hippocampus-related brain changes on CRAI. Since it is not clear whether hippocampus-related brain changes cause depression and anxiety or whether affective changes are caused by the diagnosis, further research should focus on understanding the cause and interacting effects of various factors, such as inflammation or individual factors, CRAI, CRCI, and brain-structural changes in BCP. The detection of fatigue as an interface between CRCI and CRAI (78), for example, could provide an insight into the interaction of treatment-related consequences.

Even though a lot of research is still needed to assess the effects of breast cancer treatment on CRCI and CRAI, the research results clearly indicate that there is a great need for these side effects to be addressed in everyday medical practice. Healthcare professionals are in the position not only to give their patients advice about the most suitable treatment approach (43, 79) but to inform them about the affective and cognitive challenges resulting from the procedure. This includes providing information about the possibility of participating in interventions.

## Conclusions

Overall, although improvements in the treatment of breast cancer cause survivorship rates to rise steadily (3, 4), treatment side effects due to hippocampus-related brain changes seem to be omnipresent across all types of therapy (52–58). CRCI due to hippocampus-related brain changes seem to be particularly relevant in cognitive domains including executive function as well as working memory, episodic memory, and prospective memory (52, 55–58). Although an interaction between hippocampus-related brain changes, CRCI, and CRAI can be hypothesized, CRAI are less reflected in current research. This is problematic because latest findings indicate that CRAI in BCP can contribute to hippocampus-related brain structural changes (56). Against this background, further hypothesis- and knowledge-based research on treatment side effects and interaction effects is required, especially by combining information received from imaging techniques and specific neuropsychological tests. Identifying the mechanisms by which hippocampus-related brain changes affect CRCI and CRAI may help to understand why those impairments even persist up to years after the end of treatment and substantiate the importance of integrating this knowledge into everyday medical practice. This could contribute to improve early detection, timely treatment, and informed therapeutic options (58) and lead to an improved quality of life, both during and after treatment, for the increasing number of women dealing with this life-threatening diagnosis.

## Author Contributions

XP, KS, PM, and PZ devised the main conceptual ideas, which have been further specified in consultation with the other authors. KS, PM, and PZ defined the databases to be searched and the inclusion and exclusion criteria for the studies. XP and AR searched the databases and performed the initial and full text screening. The studies were read by all authors and criteria for sorting and summarizing the content of the studies were provided, which were then extracted from the studies by XP and KS. The manuscript was written by XP and KS, with the help of AR. After all authors had discussed the results and commented on the manuscript, it was further compressed and specified in content by XP, AR, and SS.

### Conflict of Interest

The authors declare that the research was conducted in the absence of any commercial or financial relationships that could be construed as a potential conflict of interest.
